# SrCo_1−x_Ti_x_O_3−δ_ perovskites as excellent catalysts for fast degradation of water contaminants in neutral and alkaline solutions

**DOI:** 10.1038/srep44215

**Published:** 2017-03-10

**Authors:** Jie Miao, Jaka Sunarso, Chao Su, Wei Zhou, Shaobin Wang, Zongping Shao

**Affiliations:** 1 Jiangsu National Synergetic Innovation Center for Advanced Materials (SICAM), State Key Laboratory of Materials-Oriented Chemical Engineering, College of Chemical Engineering, Nanjing Tech University, No. 5 Xin Mofan Road, Nanjing 210009, P.R. China; 2 Faculty of Engineering, Computing and Science, Swinburne University of Technology, Jalan Simpang Tiga, 93350 Kuching, Sarawak, Malaysia; 3 Department of Chemical Engineering, Curtin University, GPO Box U1987, Perth, WA 6845, Australia

## Abstract

Perovskite-like oxides SrCo_1−x_Ti_x_O_3−δ_ (SCT_x_, x = 0.1, 0.2, 0.4, 0.6) were used as heterogeneous catalysts to activate peroxymonosulfate (PMS) for phenol degradation under a wide pH range, exhibiting more rapid phenol oxidation than Co_3_O_4_ and TiO_2_. The SCT_0.4_/PMS system produced a high activity at increased initial pH, achieving optimized performance at pH ≥ 7 in terms of total organic carbon removal, the minimum Co leaching and good catalytic stability. Kinetic studies showed that the phenol oxidation kinetics on SCT_0.4_/PMS system followed the pseudo-zero order kinetics and the rate on SCT_0.4_/PMS system decreased with increasing initial phenol concentration, decreased PMS amount, catalyst loading and solution temperature. Quenching tests using ethanol and tert-butyl alcohol demonstrated sulfate and hydroxyl radicals for phenol oxidation. This investigation suggested promising heterogeneous catalysts for organic oxidation with PMS, showing a breakthrough in the barriers of metal leaching, acidic pH, and low efficiency of heterogeneous catalysis.

Organic compounds containing wastewaters that are discharged from households and industries may produce hazardous impacts on human health and the environment. In recent years, this concern together with the limited clean water resources have led to the development of efficient remediation techniques for removal of the organic contaminants from wastewaters^1^. Currently, the removal of organic contaminants *via* advanced oxidation processes (AOPs) relies upon the generation of highly reactive species with high redox potentials^2^,^3^. Hydroxyl radical (·OH, E_0_ = 1.8–2.7 V) is the most commonly used species in the Fenton reaction which demonstrates a high efficiency to oxidize organic compounds^4^. Nonetheless, some AOPs to generate hydroxyl radicals, such as Fenton reaction, ozonation and UV radiation, give rise to several drawbacks. Fenton reaction needs to be at acidic condition (pH of 3–4), production of large amount of sludge and leaching of metal ions^5^,^6^ while ozonation and UV radiation require high energy inputs^7^,^8^. Sulfate radicals (SO_4_·^−^, E_0_ = 2.5–3.1 V) represent another active species that shows higher oxidation potential and longer half-life than hydroxyl radicals and they have been used to oxidize organic compounds such as phenol and its derivatives due to no limitation of reaction condition, faster reaction rate and lower energy requirements^9^,^10^,^11^,^12^.

The generation of sulfate radicals can be initiated via the activation of peroxymonosulfate (PMS) by photocatalysis^13^, transition metal catalysis^14^, and electrochemistry^15^. In particular, tradition metal activation, which involves homogeneous and heterogeneous catalysis, has been considered as the most effective process^16^,^17^. The associated metal ion leaching problems, however, limit the applicability of tradition metal catalysts, because of the hazardous risk of the metal ions to human health and the environment. The Fenton-like heterogeneous catalyst systems are more practical for phenol oxidation involving PMS activation. Co/PMS process has been reported to be highly effective for organic compound degradation despite the drawbacks of utilizing Co ions (for their subsequent pollution risk to the environment)^18^,^19^,^20^,^21^. Accordingly, several Co-based heterogeneous catalysts, e.g., Co oxides^22^, supported Co^23^,^24^, Co exchanged zeolites^25^,^26^, and bimetallic Co-based oxides^27^,^28^, have been studied for PMS activation, while the issue has not been fully resolved.

A perovskite oxide has a chemical formula of ABO_3_ in which alkaline earth metals or rare-earth metals can be incorporated into the A-site while transition metals can be incorporated into the B-site. The presence of oxygen vacancies from oxygen non-stoichiometry in concomitant with the redox couple from the transition metals endow such perovskite oxides mixed (oxygen) ionic-electronic conductivity and reduction-oxidation (redox) properties^29^, which are beneficial for applications in solid oxide fuel cells, oxygen permeation membranes, metal-air batteries, and electrochemical water splitting^30^,^31^,^32^,^33^. Taran *et al*. reported the catalytic activity of LaCuO_3_ for wet peroxide oxidation of phenol, which indicates the potential applicability of perovskite oxides for catalytic oxidation^34^. Nevertheless, perovskite oxides have been barely used in AOPs for PMS activation^35^.

In this work, we reported that Ti-doped SrCoO_3−δ_ (SCO) perovskites can effectively activate PMS for generation of sulfate radicals. The synthesized SrCo_1−x_Ti_x_O_3−δ_ (SCT_x_, x = 0, 0.1, 0.2, 0.4, and 0.6), in fact, demonstrated a higher catalytic activity with respect to Co_3_O_4_. In terms of the minimum Co ion leaching, SCT_0.4_ is viewed as the best catalyst which also showed a high catalytic activity for phenol oxidation. The effects of initial phenol concentration, PMS amount, catalyst loading, and solution temperatures on SCT_0.4_/PMS system for phenol degradation were evaluated. Furthermore, in figuring out the most plausible mechanism of activation, we also evaluated the effect of solution pH and studied the oxidation kinetics in the presence of ethanol and tert-butyl alcohol that react distinctly to sulfate and hydroxyl radicals. It was found that SCT_0.4_/PMS presented higher efficiencies in organic degradation at high pH, which can overcome the common problems of Fenton reaction and provide a promising application for real wastewater treatments under alkaline conditions.

## Experimental

### Synthesis of perovskite oxide catalysts

A combined ethylenediaminetetraacetic acid-citric acid (EDTA-CA) complexing sol-gel route^36^ was used to synthesize the perovskite oxide catalysts, SrCo_1−x_Ti_x_O_3−δ_ (SCT_x_, x = 0.1, 0.2, 0.4, and 0.6). Detailed information is presented in supplementary data.

### Catalyst characterizations

The properties of catalysts were investigated by various techniques, which are detailed in supplementary data.

### Catalyst tests

Batch oxidation tests of phenol degradation were carried out in a 1 L glass vessel containing 500 mL of phenol solution (with a concentration of 20 ppm), SrCo_0.6_Ti_0.4_O_3−δ_ (SCT_0.4_, 0.1 g L^−1^) and peroxymonosulphate (PMS, 2 g L^−1^) under continuous stirring (250 rpm) in a temperature-controlled water bath at 25 °C. Detailed information is presented in supplementary data.

## Results and Discussion

### Characterizations and catalytic of perovskite oxides

**Figure 1:**
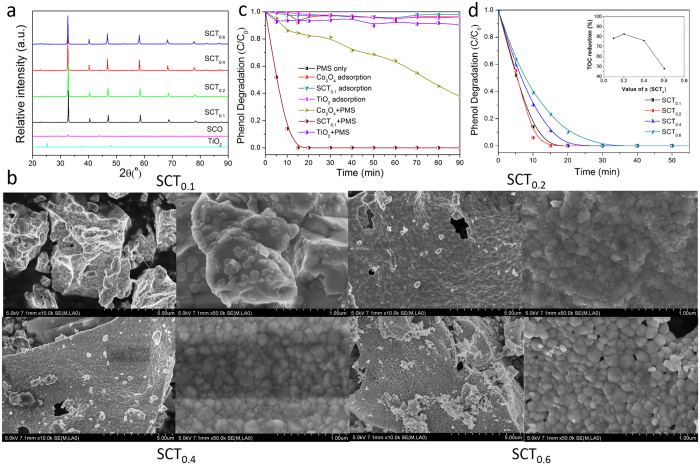
Characterization of synthesized SCT_x_ (x = 0, 0.1, 0.2, 0.4, and 0.6) and TiO_2_ catalysts and their catalytic performances on phenol degradation with PMS activation. (**a**) XRD; (**b**) SEM; (**c**) Time-dependent phenol degradation profiles for Co_3_O_4_, TiO_2_, and SCT_0.1_ in the presence or absence of peroxymonosulfate (PMS) (Adsorption occurred in the absence of PMS while oxidation occurred when both oxide catalyst and PMS were present); and (**d**) Time-dependent phenol degradation profiles for different oxide catalysts, SCT_x_ (x = 0.1, 0.2, 0.4, and 0.6) in the presence of PMS; Inset – Total organic carbon reduction (in %) during phenol degradation for 2 h; Reaction conditions: C_0_ (initial phenol concentration) = 20 mg L^−1^; catalyst loading = 0.1 g L^−1^; PMS loading = 2.0 g L^−1^; and temperature = 25 °C.

The phase structure of the SCT_x_ (x = 0.1, 0.2, 0.4, and 0.6) catalysts calcined at 1000 °C for 6 h was obtained by powder X-ray diffraction (XRD) and their patterns are shown in Fig. 1(a). The characteristic peaks of all samples can be indexed according to the primitive cubic perovskite lattice with a space group *Pm*
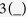
*m* (#221)^30^. In Fig. 1(a), SCT_x_ have stronger intensity of diffraction peaks of perovskites than SCO, which presented higher stability of perovskite-type structure. Compared with the XRD patterns of SCO and TiO_2_ (see Fig. 1(a)), SCT_x_ (x = 0.1, 0.2, 0.4, 0.6), which exhibited the single phase, at varying ratio of Ti addition will not change the perovskite-type structure. The characteristic peaks of all perovskites overlap each other, suggesting almost identical lattice parameters for all the perovskites. With increasing Ti content, the most intense characteristic peak at around 2θ of 33° appears to shift slightly toward the lower angles. This indicates an expansion of SCT_x_ lattice, due to the partial substitution of Co by Ti (Note that Co^3+^(VI) HS (high spin) has an ionic radius of 0.61 Å while Ti^4+^(VI) has an ionic radius of 0.75 Å)^37^.

The particulate morphology of SCT_x_ (x = 0.1, 0.2, 0.4, and 0.6) was then carried out by SEM and the typical images are shown in Fig. 1(b). Ti-doped SrCoO_3−δ_ samples clearly experienced significant agglomeration during calcination at 1000 °C for 6 h, which had a large size of 1–5 μm. An increase in Ti content (or equivalently, the increase in x) somewhat led to the loosening of grain agglomeration (Fig. 1(b)). Relatively identical nano-sized grains of 10–20 nm are nonetheless observed for all four samples (See Fig. 1(b)). Nitrogen sorption furthermore reveals an increasing Brunauer-Emmett-Teller specific surface area (S_BET_) with increasing Ti content, i.e., the surface area increased from 0.4 m^2^ g^−1^ for x = 0 to 1.2 m^2^ g^−1^ for x = 0.6 (Supplementary Table S1) that supports the SEM observation.

To explore the capability of perovskite oxides for removing phenol, the toxic organic with stable structure, adsorption and/or oxidation of phenol in the presence or absence of different oxide catalysts, with or without PMS were tested, as shown in Fig. 1(c). To provide baselines for the catalytic performance comparison, oxide catalyst self-adsorption and PMS self-oxidation were firstly checked. PMS clearly could not induce any noticeable phenol degradation in the absence of catalysts. In this case, less than 5% of phenol was removed during 90 min. Adsorption of phenol on Co_3_O_4_, TiO_2_, or SCT_0.1_ was the only possible mechanism in the absence of PMS. Such adsorption may not translate to effective phenol removal given the low surface areas of these oxides. Accordingly, less than 5% of phenol was removed during the 90 min-period which implies negligible phenol adsorption on these oxides.

Oxidations occurred when both oxide catalysts (i.e., Co_3_O_4_, TiO_2_, or SCT_0.1_) and PMS were present, in which phenol degradations become more pronounced. These oxidations are related to the activation of PMS to generate sulfate and hydroxyl radicals that can degrade phenol. SCT_0.1_/PMS system showed the largest catalytic activity, degrading the phenol in 15 min. For Co_3_O_4_ and TiO_2_, however, only ~60% and ~10% of the phenol were degraded in 90 min, respectively. The catalytic performance thus increased in an order of TiO_2_ < Co_3_O_4_ < SCT_0.1_ (see Fig. 1(c)). Moreover, the total organic carbon (TOC) analysis showed that about 78% of phenol could be mineralized to CO_2_ after 2 h-oxidation using SCT_0.1_/PMS system. And compared with Co_3_O_4_ (14.4 m^2^g^−1^) and TiO_2_ (9.5 m^2^g^−1^), SCT_0.1_ (0.4 m^2^g^−1^) has the lowest specific surface area (Supplementary Table S1), which all investigated that SCT_0.1_ has the excellent catalytic performance for phenol oxidation with PMS activation.

The catalytic performances of SCT_x_ (x = 0.1, 0.2, 0.4, and 0.6) was also investigated in the PMS activation for the oxidative degradation of phenol (Fig. 1(d)). SCT_0.2_ showed the highest catalytic degradation efficiency. Increasing surface area for the perovskite with increasing Ti content did not appear to well correlate with the catalytic improvement for phenol oxidation (Supplementary Table S1). The general trend (Fig. 1(d)) indicates a decreasing rate of phenol oxidation with increasing titanium (Ti) content on the perovskite although SCT_0.6_ can still be considered as a highly active catalyst given its capability to completely oxidize phenol within 45 min in the presence of PMS. The leaching of metal ions from the perovskite matrix to the solution was evaluated using ICP-AES. Co concentration in the filtrate varies from 3.1 to 5.4 mg L^−1^ (at pH of 3–4) which implies substantial leaching out of Co from SCT_x_ in acidic condition. Co concentration appears to decrease with increasing Ti content in the perovskite. On the other hand, Ti leaching is not as obvious as Co considering its relatively lower concentration, i.e. below 1.0 mg L^−1^ in all perovskite cases. Therefore, it is a trade-off to obtain both maximized catalytic activity and minimized Co leaching. SCT_0.4_ showed a similar Co concentration of 3.1 mg L^−1^ to SCT_0.6_, indicating either SCT_0.4_ or SCT_0.6_ could be chosen. The TOC analysis for SCT_x_ (x = 0.1, 0.2, 0.4, and 0.6) showed the maximum TOC reduction on SCT_0.2_ (82% TOC reduction) after 5 h phenol oxidation (Inset of Fig. 1(d)). SCT_0.4_ could reduce about 77% of TOC over this duration. So given their identical Co leaching level and higher catalytic activity of SCT_0.4_, more tests were then focused on SCT_0.4_.

### The effects of operating conditions on SCT_0.4_/PMS system and reusability of SCT_0.4._

**Figure 2:**
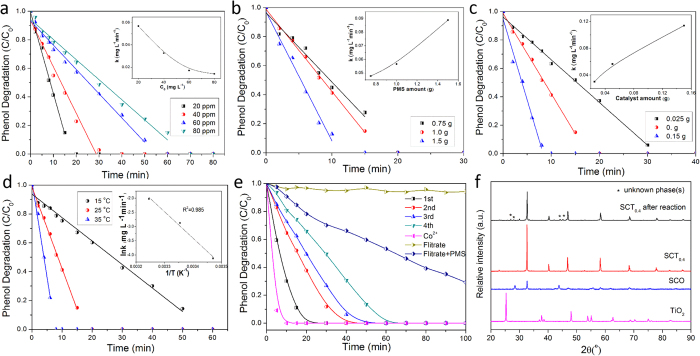
Factorial effects on the catalytic degradation of phenol in SCT_0.4_/PMS system and recyclability of SCT_0.4_-activated PMS. (**a**) initial phenol concentration; (**b**) PMS amount; (**c**) the catalyst amount; (**d**) the reaction temperature; (**e**) recyclability of SCT_0.4_-activated PMS and phenol degradation by homogeneous catalysis (Co^2+^ same molar amount of Co(NO_3_)_2_ with PMS) and (**f**) XRD of TiO_2_, SrCoO_3−δ_ (SCO),SCT_0.4_ before and after phenol oxidation for 2 h. Reaction conditions: C_0_ (initial phenol concentration) = 20 mg L^−1^; catalyst loading = 0.1 g L^−1^; PMS loading = 2.0 g L^−1^; reaction volume = 500 mL and temperature = 25 °C.

As phenol was efficiently degraded by SCT_0.4_-activated PMS, we investigated SCT_0.4_/PMS system for phenol under different operating conditions, such as initial phenol concentration, PMS amount, catalyst loading and reaction temperature. Figure 2(a) displays the kinetic dependence of phenol oxidation on initial phenol concentration (C_0_). The inset of Fig. 2(a) further shows the dependence of the reaction rate constant (k) for phenol oxidation on the initial phenol concentration (C_0_). Phenol oxidation at 100% is attained within 30 min when the initial phenol concentration is less than 40 mg L^−1^, while the oxidations at only 71% and 64% are attained for the initial phenol concentrations of 60 mg L^−1^ and 80 mg L^−1^, respectively. Considering a fixed amount of catalyst, Fernandez *et al*.^21^. and Shulkla *et al*.^38^. provided a pseudo-zero-order kinetics of phenol removal, which is presented by the following equation:


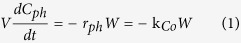


where k_Co_ is the apparent zero order constant, C_ph_ is the phenol concentration at any instant time (t), V is the volume of reactor, and W is the mass of catalyst. Therefore, the mathematical model of the degradation profile in Fig. 2(a,b,c,d) is induced by the integration of the above Equation (1). Shukla *et al*. also studied the kinetics of phenol oxidation by heterogeneous activation of PMS with different Co catalysts and reported that zero-order or first-order kinetics may occur depending on the catalysts used^24^,^25^,^38^,^39^. Table 1 shows the rate constants and the regression coefficients followed the pseudo-zero-order kinetics at four different initial phenol concentrations. The reaction rate decreased with the increasing initial phenol concentration. The reasonably good fittings are indicated by the high values of regression coefficients (R^2^ > 0.97).

The PMS amount also shows a significantly effect on kinetic phenol oxidation in Fig. 2(b). Higher amount of PMS led to a higher oxidation rate which translates to reduced time for complete oxidation of PMS. For example, when double amount of PMS was added, i.e., 1.5 g of PMS relative to 0.75 g of PMS, phenol oxidation could be completed within 10 min instead of 20 min.

Besides, the phenol oxidation kinetic dependence on the catalyst loading was probed as displayed in Fig. 2(c). Likewise, an increase in catalyst loading also correlates with the increase in the reaction rate. This is consistent with the expected increase of the active sites (Co) for phenol oxidation that enhances the generation of sulfate and hydroxyl radicals^24^.

Phenol degradation by SCT_0.4_-activated PMS was conducted at different reaction temperatures to further study the catalytic activity of SCT_0.4_ for the activation of PMS. Figure 2(d) shows phenol oxidations at three different solution temperatures, 15, 25, and 35 °C while complete phenol oxidations were attained within 50, 20, and 10 min, respectively. Such an increase in reaction rate with increasing solution temperature highlights the endothermic nature of the phenol oxidation on SCT_0.4_/PMS system. The inset of Fig. 2(d) displays the thermal-dependent Arrhenius representation of the pseudo-zero order reaction rate constant (k) for phenol oxidation. By using the pseudo-zero order kinetic equation and the Arrhenius equation, the activation energy (E_a_) for the oxidation process was obtained as 77.5 kJ mol^−1^ (see Table 2). The absence of perovskite data for PMS activation means that the activation energies for phenol oxidation on SCT_0.4_/PMS system should be compared to phenol oxidations on the other more widely studied catalyst systems (Table 2). As we all know, one of critical factors influencing on the reaction activation energy is catalysts. Therefore, Table 2 showed that the phenol oxidation with different catalysts have different activation energies. Co-SiO_2_ and Co-ZSM-5, in particular, showed the closest activation energies to SCT_0.4_/PMS system. Co/AC and Co/Fly-ash exhibited the lower activation energy.

As a heterogeneous catalyst, the recyclability of SCT_0.4_ is critical for practical implication. Figure 2(e) depicts the performance of the recycled SCT_0.4_ catalyst for phenol degradation. The catalyst was recovered using filtration followed by mild washing with de-ionized water while the filtrate was stored for further analysis. The reused SCT_0.4_ catalyst can still catalyze complete phenol oxidation within 60 min after three subsequent recycles. It showed more pronounced decrease in the activity after the first recycle followed by less decrease in activities in the subsequent recycles. TOC analysis showed that the TOCs were reduced by 76.2% and 75.3% during the first 2 h period of phenol oxidations in the first and second runs, respectively (see Supplementary Table S3). Increasing the reaction time up to 6 h can improve the TOC reduction to above 80% even after the second recycle (see Supplementary Table S3).

The leaching of Co^2+^ from perovskite matrix was inevitable given the necessary long term contact and constant mixing between the perovskite catalysts and the aqueous phase. We also tested phenol oxidation using Co^2+^/PMS system in an analogous mole amount to SCT_0.4_ as well as phenol oxidation using the filtrate from the previous SCT_0.4_/PMS system of the first run. The phenol oxidation on Co^2+^/PMS system was more rapid than the phenol oxidation on filtrate/PMS. This suggests that cobalt leaching did occur but it was at a much lower rate reflected by its low reaction rate.

We also did some characterizations of the reused SCT_0.4_ to study its stability. Figure 2(f) shows the powder XRD patterns of the catalysts before and after the phenol oxidation for 2 h to check the possible changes in the phase compositions. The main perovskite phase of SCT_0.4_ was retained although unknown phase(s) appeared afterwards, which are likely due to decomposition of the perovskite phase caused by metal leaching^40^ and carbonate adsorption on the solid surface^34^.

**Figure 3:**
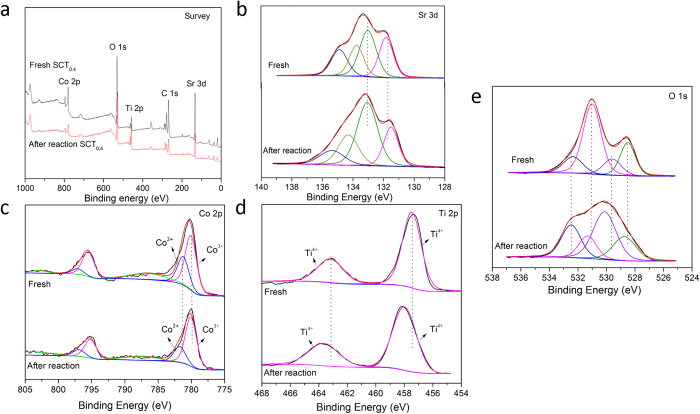
X-ray photoelectron spectra of SCT_0.4_ before and after phenol oxidation for 2 h; (**a**) survey scan, (**b**) High resolution Sr 3d spectra, (**c**) High resolution Sr 3d spectra, (**d**) High resolution Ti 2p spectra, and (**e**) High resolution O 1 s spectra.

The changes in the surface composition of the fresh and used SCT_0.4_ catalysts were further evaluated using X-ray photoelectron spectroscopy (XPS). Figure 3(a) displays the survey scan on SCT_0.4_ surface, evidencing the presence of Sr, Co, Ti, and O elements. The high resolution Sr 3d XPS spectra for the fresh and used SCT_0.4_ catalysts (Fig. 3(b)) further reveal the formation of four peaks at 134.9 eV, 133.8 eV, 133.0 eV, and 131.8 eV. Sr 3d_3/2_ components are located at higher binding energy (BE) while 3d_5/2_ components are located at lower BE^41^. Accordingly, Sr 3d_5/2_ peak at 131.8 eV and Sr 3d_3/2_ peak at 133.0 eV correspond to Sr in the perovskite phase^42^. Sr 3d peak at approximately 133.8 eV, on the other hand, is likely related to the strontium carbonate on the surface^34^,^43^,^44^.

Figure 3(c) further shows the high resolution Co 2p XPS spectra for the fresh and used SCT_0.4_ catalysts. The Co 2p spectra contain 2p_1/2_ and 2p_3/2_ components. The peaks at 786.4 eV and 802.9 eV were the additional satellite peaks for Co 2p_1/2_ and Co 2p_3/2_, respectively. The peak located at BE of 781.2 eV (Co 2p_3/2_) represents Co^3+^ while the peak located at BE of 780.1 eV (Co 2p3/2) represents Co^2+^, consistent with the literature^27^,^43^,^44^. The peaks corresponding to Co^3+^ and Co^2+^ can be deconvoluted to determine their peak areas. Accordingly, Co^3+^ to Co^2+^ ratio increased from 1.93 to 2.93 after the phenol oxidation for 2 h. This may indicate the activity of Co^2+^/Co^3+^ redox couple (SCT_0.4_-Co(II)/SCT_0.4_-Co(III)) in catalyzing the phenol oxidation in the presence of PMS. However, the average cobalt oxidation states were calculated approximately to be 2.71 and 2.73 in the fresh and used SCT_0.4_, respectively. The results suggest that the cobalt oxidation states in the phase of perovskite were almost unchanged, which indicates the stability of SCT_0.4_ in the catalytic process of phenol oxidation, and the metal leaching is likely to be mainly caused by the perovskite oxides dissolved in the acidic solution.

The high resolution Ti 2p XPS spectra for the fresh and used SCT_0.4_ catalysts are depicted in Fig. 3(d) which features two peaks for 2p_3/2_ and 2p_1/2_ components at 457.5 eV and 463.2 eV, respectively; both of which can be assigned to Ti^4+^. Despite the slight shift in the binding energy, Ti ions in perovskite existed as Ti^4+^ after the phenol oxidation for 2 h.

Figure 3(e) shows the high resolution O 1 s XPS spectra for the fresh and used SCT_0.4_ catalysts. The oxygen O 1 s peak can be deconvoluted into four sub-peaks. The peak at BE of approximately 528.4 eV represents the lattice oxygen within the perovskite oxides^4^. The other two peaks at BE of approximately 529.7 eV and 531.2 eV can be ascribed to the chemisorbed oxygen which co-existed in the form of O_2_^2−^ and O^−^ at the surface. The peak at the highest BE of around 532.6 eV, on the other hand, can be assigned to O_H_ from the hydroxyl groups or carbonates^27^,^42^,^44^. Carbonates may be generated during the complex adsorption process of organic compounds or during the oxidation reactions that involves the formation of carbon-based intermediates^34^, likely corresponding to the unknown phase(s) in the XRD of Fig. 2(f). Therefore, the carbonate adsorption on the surface of SCT_0.4_ after reaction may cause the deactivation of SCT_0.4_ during the subsequent reuses.

### The effects of initial pH on SCT_0.4_/PMS system

**Figure 4:**
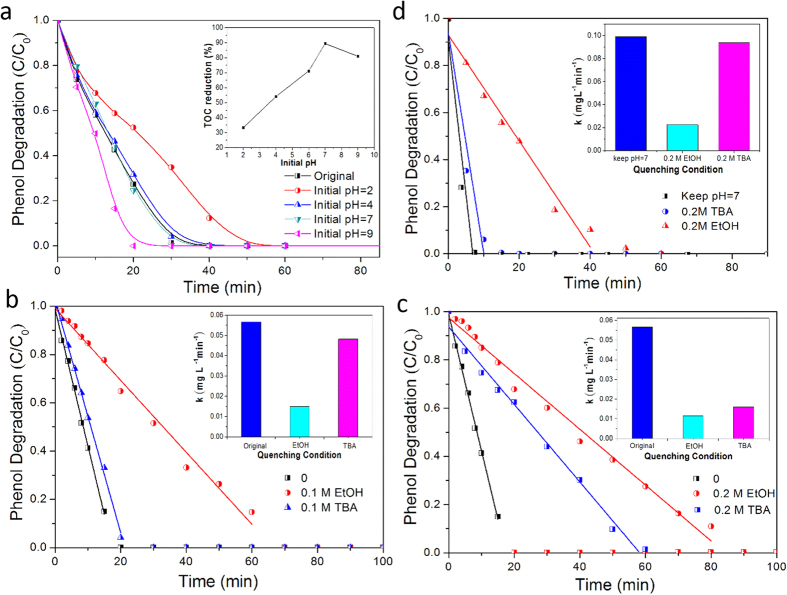
Effects of different initial solution pH and radical inhibitors on catalytic degradation of phenol in SCT0.4/PMS system. (**a**) different initial solution pH (pH range was from 2 to 9), inset - Total organic carbon reduction (%) during phenol degradation for 5 h as a function of pH; The oxidation kinetics dependence (**b**) and (**c**) in the presence of the quenching agents (ethanol – EtOH and tert-butyl alcohol TBA) at different concentrations (0.1 M and 0.2 M); and (**d**) The oxidation kinetics dependence when the neutral condition (pH of 7) was maintained throughout the oxidation course; Inset of (**b,c,d**) – Reaction rate constant in the absence or the presence of EtOH or TBA; Reaction conditions: C_0_ (initial phenol concentration) = 20 mg L^−1^; catalyst loading = 0.1 g L^−1^; PMS loading = 2.0 g L^−1^; reaction volume = 500 mL and temperature = 25 °C.

For the aqueous reaction of PMS activation, initial pH of reaction solution is an important factor to influence the interfacial interactions between PMS, organics and catalysts^45^ and was further investigated. Figure 4(a) presents the phenol oxidation on SCT_0.4_/PMS system at different initial solution pH. The pH of the original phenol solution is around 6. We used 0.1 M H_2_SO_4_ or 0.1 M KOH to adjust the initial solution pH. Relative to the neutral condition (pH of 7), higher acidity lowers the oxidation rate while higher alkalinity is in the opposite way. The effect of pH on the phenol degradation rate was attributed to the concentration change of the active radicals in the solution. When the pH is below 7, OH^−^ ions react with H^+^ ions in neutralization reaction to generate water. This essentially inhibits the two primary reactions that generate hydroxyl radicals (·OH, See Equations (5) and (6) which will be discussed below). Likewise, increasing the amount of OH^−^ served to shift these reaction equilibria to the products, thus favoring the generation of hydroxyl radicals.

It is interesting that the TOC reduction (after 2 h) trend did not exactly follow the phenol oxidation rate trend. Maximum TOC reduction was observed at the neutral condition (pH of 7) (See inset of Fig. 4(a)). The fact that the TOC reduction for the initial pH = 9 was actually lower than that at the initial pH = 7 can be rationalized in terms of the derived phenoate (that was formed in an alkaline condition) which may slow down the reactivity of phenol and reaction intermediates in oxidation processes^46^.

The initial solution pH also showed a significant effect on metal ion leaching. The concentration of metal ions in the solution sharply decreased by increasing initial solution pH. Taking Co leaching as an example, with respect to the initial pH = 6 where no H_2_SO_4_ or KOH was added, the initial pH = 6 resulted in the lower concentration of cobalt ions in the solution (C_Co_ = 3.1 mg L^−1^). Increasing the pH further as represented by the initial pH = 9 led to an even lower concentration of cobalt ions in the solution (C_Co_ = 2.2 mg L^−1^).

Maintaining the solution pH neutral (pH of 7) by constant pH monitoring and periodic addition of KOH) throughout the oxidation process minimized the Co leaching problem. Compared to the initial pH = 6, much lower concentration of cobalt ions in the solution was obtained (C_Co_ = 1.3 mg L^−1^). This additionally resulted in a more rapid phenol oxidation than the initial pH = 9 (Compare Fig. 4(a) with Fig. 4(d) and note the different y-axis representations). By keeping the solution pH at 7 throughout the oxidation process, complete phenol oxidation can be achieved within 10 min as opposed to 20 min required for the initial pH = 9. The preservation of neutral pH during the oxidation course clearly optimized the phenol oxidation performance on SCT_0.4_/PMS system.

### Catalytic mechanism

Several studies have reported that PMS activation using metal-based catalysts involve of two key active radicals, i.e., sulfate radicals (SO_4_·^−^) and hydroxyl radicals (·OH)^10^,^47^,^48^,^49^. To reveal the phenol degradation mechanism in SCT_0.4_/PMS system, different radical inhibitors, ethanol (EtOH) and tert-butyl alcohol (TBA), were employed to evaluate the reaction. It is worth noting that EtOH (with α-H) readily reacts with both SO_4_·^−^ and ·OH whereas TBA (without α-H) reacts mainly with ·OH and is inert to SO_4_^40^,^50^,^51^. Figure 4(b,c,d) confirms that the phenol degradation rate was reduced with the presence of these radical inhibitors and their increasing concentrations. The presence of 0.2 M EtOH led to k of 0.012 mg L^−1^ min^−1^, lower than k of 0.016 mg L^−1^ min^−1^ obtained in the presence of 0.2 M TBA in the system. This suggests that both SO_4_·^−^ and ·OH were generated in the PMS activation process using SCT_0.4_, where normally more SO_4_·^−^ than ·OH formed, as reported in several previous works^27^,^52^,^53^. Moreover, Fig. 4(d) reveals that upon maintaining neutral condition (pH = 7) throughout the oxidation course, more rapid phenol oxidation was obtained than that in the original oxidation case where the pH was not controlled. Relative to the original case, the phenol oxidation rate at the neutral condition increased by 1.95 times and 5.84 times for the tests with 0.2 M EtOH and 0.2 M TBA, respectively. This is likely due to the increased active radical species at such neutral condition. In the insert of Fig. 4(c,d), the presence of 0.2 M TBA at the neutral condition k shows more higher value than that without pH control, whereas the presence of 0.2 M EtOH keeps the similar phenol oxidation rate, which could be explained by more SO_4_·^−^ generated than ·OH with pH control at 7, for that TBA reacts mainly with ·OH, but EtOH readily reacts with both them.

Our aforementioned characterization results and literature survey led us to propose the most plausible Fenton-like mechanism of phenol oxidation on SCT_0.4_/PMS system as follows:


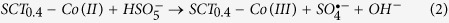



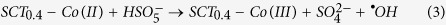



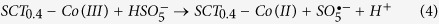



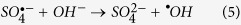



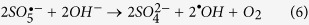



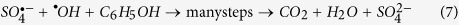


SO_4_·^−^ and ·OH are initially generated simultaneously by the activation of PMS on SCT_0.4_ (Equations (2) and (3)). Then a majority of the produced SO_4_·^−^ radicals react with phenol rapidly, due to their higher potential (E_0=_2.5–3.1 V)^9^. Moreover, some ·OH radicals also participate in the oxidation at a slower rate, leaving ·OH radicals as the only active species after all SO_4_·^−^ has been consumed (Equation (7)). The Co^2+^/Co^3+^ redox couple on SCT_0.4_ (SCT_0.4_-Co(II)/SCT_0.4_-Co(III)) reacts in cycles with PMS to generate the reactive oxygen species (Equations (2), (3), and (4)). Moreover, the generated SO_5_·^−^ radicals (Equation (4)), which have relatively low potential (E_0_ = 1.1 V)^54^, react with OH^−^ ions to produce ·OH radicals that completely oxidize (mineralize) phenol (Equation (7)).

## Conclusions

We have demonstrated that SrCo_1−x_Ti_x_O_3−δ_ (x = 0.1, 0.2, 0.4, and 0.6) perovskite oxides exhibit a fast catalytic activity for phenol oxidation in the presence of PMS in a wide pH range. Powder XRD patterns showed cubic perovskite lattice and its stable structure in aqueous reaction. The catalytic performance for phenol oxidation presented an order of SCT_0.6_ < SCT_0.4_ < SCT_0.1_ < SCT_0.2_. The operating conditions can be adjusted to increase the phenol oxidation rate by increasing PMS amount, catalyst loading, and initial pH. Under neutral and alkaline conditions, SrCo_1−x_Ti_x_O_3−δ_/PMS presented effective degradation efficiency of aqueous contaminants with less metal leaching and higher TOC reduction rate, which is better than Fenton-like systems and provides promising application for organic oxidation with sulfate radicals.

## Additional Information

**How to cite this article**: Miao, J. *et al*. SrCo_1−x_Ti_x_O_3−δ_ perovskites as excellent catalysts for fast degradation of water contaminants in neutral and alkaline solutions. *Sci. Rep.* **7**, 44215; doi: 10.1038/srep44215 (2017).

**Publisher's note:** Springer Nature remains neutral with regard to jurisdictional claims in published maps and institutional affiliations.

## Supplementary Material

Supplementary Information

## Figures and Tables

**Table 1:** Kinetic parameters of phenol oxidation on SCT_0.4_/PMS system at different initial phenol concentrations.

Initial phenol concentration (ppm)	Rate constant (mg L^−1^min^−1^)	R^2^ of k
20	0.056	0.997
40	0.032	0.975
60	0.017	0.986
80	0.014	0.985

**Table 2:** Activation energies for phenol oxidation in the presence of PMS on several Co-based catalysts.

Catalyst	Activation energy (kJ mol^−1^)	References
Co/Fly-ash	66.3	23
Co-SiO_2_	61.7–75.5	24
Co/AC	59.7	25
Co-ZSM-5	69.7	38
Co_3_O_4_	66.2	47
SrCo_0.6_Ti_0.4_O_3−δ_	77.5	This work
