# Supplementary material for: SrCo1−xTixO3−δ perovskites as excellent catalysts for fast degradation of water contaminants in neutral and alkaline solutions
Source: Sci Rep. 2017 Mar 10;7:44215. doi: 10.1038/srep44215 (PMC5345050; doi:10.1038/srep44215)
Supplement: Supplementary Information [file srep44215-s1.pdf]

Supplementary Data

$SrCo_{1-x}Ti_xO_{3-\delta}$ perovskites as excellent catalysts for fast degradation of water contaminants in neutral and alkaline solutions

Jie Miao[a], Jaka Sunarso[b], Chao Su[c], Wei Zhou[a,*], Shaobin Wang[c,*], Zongping Shao[a,c]

[a]*Jiangsu National Synergetic Innovation Center for Advanced Materials (SICAM), State Key Laboratory of Materials-Oriented Chemical Engineering, College of Chemical Engineering, Nanjing Tech University, No.5 Xin Mofan Road, Nanjing 210009, P.R. China*

[b]*Faculty of Engineering, Computing and Science, Swinburne University of Technology, Jalan Simpang Tiga, 93350 Kuching, Sarawak, Malaysia*

[c]*Department of Chemical Engineering, Curtin University, GPO Box U1987, Perth, WA 6845, Australia*

AUTHOR INFRMATION

* To whom correspondence should be addressed:

Name: Wei Zhou; Phone: +86 147 5106 9990; Fax: +86 147 5106 9990; E-mail: *zhouwei1982@njtech.edu.cn*

Name: Shaobin Wang; Phone: +61 8 9266 3776; Fax: +61 8 9266 2681; E-mail: *shaobin.wang@curtin.edu.au*

Synthesis of perovskite oxide catalysts

Taking $SrCo_{0.9}Ti_{0.1}O_{3-\delta}$ ($SCT_{0.1}$) as an example, tetrabutyl titanate and CA (in 1 to 5 molar ratio) were mixed in de-ionized water at 80 °C under stirring to form a clear and transparent aqueous solution. Then, stoichiometric amounts of $Sr(NO_3)_2$ and $Co(NO_3)_2{\cdot}9H_2O$ powders were dissolved into this solution. EDTA and CA were added sequentially for total metal nitrates: EDTA: CA at a fixed molar ratio of 1: 1: 2. The pH of the resulting solution was then adjusted to around 6-7 by adding ammonia ($NH_3{\cdot}H_2O$). The resultant solution was later heated to evaporate the water, forming a gel. This gel was combusted at 250 °C for 5 h to give a perovskite

precursor and then further calcined at 1000 °C for 6 h, resulting in $SCT_{0.1}$ powder.

Catalyst characterizations

The phase compositions of the perovskite oxide catalysts were probed using X-ray diffraction (XRD, Bruker D8 Advance, Germany) with filtered Cu-Kα radiation operated at 40 kV and 40 mA. The 2θ scan range is from 20 to 90°. The morphologies of the perovskite oxide powders were obtained using field-emission scanning electron microscopy (FE-SEM, Hitachi S-4800, Japan). Surface elemental analysis was performed by X-ray photoelectron spectroscopy (XPS, PHI5000, Japan) using a VersaProbe spectrometer equipped with an Al-Kα X-ray source. The Brunauer-Emmett-Teller specific surface area was measured using nitrogen sorption analysis (Quanta Autosorb-iQ, USA).

Catalyst testing

The reaction was performed for 1.5 h. At every fixed time interval, 1 mL sample was withdrawn by a syringe and filtered through a 0.45 μm filter film. The filtered solution was then drawn into a syringe and injected into a vial for the subsequent analysis. Approximately 0.5 mL of methanol was added as a quenching reagent. Adsorption experiments were carried out using the identical reaction conditions without the addition of PMS.

Phenol concentration was analyzed using high-performance liquid chromatography (HPLC, Agilent 1260, USA) with a UV detector at λ = 270 nm. A SB-C18 column was used to separate the organic components. The mobile phase was consisting of 60 vol.% of CH_3OH and 40 vol.% of water with a flow rate of 1.0 mL min^{-1}. For a few selected tests, total organic carbon (TOC) was determined by a TOC analyzer (Multi N/C 3100, Germany). For the TOC measurements, approximately 10 mL of reaction sample was extracted at the beginning and end of the experiment. Then, 10 mL of 3 M sodium nitrite solution was injected into the sample to quench the reaction. The amount of leached strontium, cobalt, and titanium were quantified using inductively coupled plasma-atomic emission spectroscopy (ICP-AES, Optima 7000 DV, Perkin-Elmer, USA) to evaluate the catalyst stability or equivalently, the metal ion leaching

from the perovskite matrix into the solution.

The effect of dissolved cobalt ions (as homogeneous component) on phenol degradation was determined using the filtrate after the reaction. The reaction was conducted in the same temperature-controlled reactor in which the filtrate, phenol, and PMS were added in their appropriate amounts, under the same conditions as the normal reaction.

For the multi-cycle tests of $SCT_{0.4}$, the used catalyst was recycled by vacuum filtration and washed with distilled water for several times after each run. The collected catalysts were dried at 60 ^{o}C prior to re-use. For radical detection, ethanol (EtOH) and tert-butyl alcohol (TBA) were used to analyze the dominant free radicals, which is also known as quenching process. Thus, the quenching tests were carried out using the different quenchers, which were added into the solution before PMS addition.

Results

Table S1. Specific surface areas of $SrCo_{1-x}Ti_{x}O_{3-\delta}$ (x =0, 0.1, 0.2, 0.4, and 0.6), Co_3O_4 and TiO_2.

Composition	S_{BET}, m^2 g^{-1}
$SrCoO_{3-\delta}$	0.4
$SrCo_{0.9}Ti_{0.1}O_{3-\delta}$	0.4
$SrCo_{0.8}Ti_{0.2}O_{3-\delta}$	0.5
$SrCo_{0.6}Ti_{0.4}O_{3-\delta}$	0.9
$SrCo_{0.4}Ti_{0.6}O_{3-\delta}$	1.2
TiO_2	9.5
Co_3O_4	14.4

Table S2. Kinetic parameters and activation energy of phenol oxidation on $SCT_{0.4}$/PMS system at different solution temperatures.

T (^oC)	Rate constant (mg $L^{-1}min^{-1}$)	R^2 of k	E_a (kJ • mol^{-1})	R^2 of E_a
15	0.016	0.988	77.5	0.985
25	0.050	0.997		
35	0.13	0.990		

Table S3. Total organic carbon (TOC) reductions (in %) during phenol oxidations on $SCT_{0.4}$/PMS system after recycle.

Cycle No.	Reaction time, h	TOC reduction
1	2	76.2%
	6	91.2%
2	2	75.3%
	6	85.5%
3	2	64.7%
	6	81.3%
